# Influence of the Acetabular Cup Material on the Shell Deformation and Strain Distribution in the Adjacent Bone—A Finite Element Analysis

**DOI:** 10.3390/ma13061372

**Published:** 2020-03-18

**Authors:** Danny Vogel, Matthias Klimek, Michael Saemann, Rainer Bader

**Affiliations:** Biomechanics and Implant Technology Research Laboratory, Department of Orthopaedics, Rostock University Medical Center, Doberaner Straße 142, 18057 Rostock, Germany

**Keywords:** modular acetabular cup, poly-ether-ether-ketone (PEEK), titanium, ceramics, ultra-high-molecular-weight polyethylene (UHMW-PE), implant deformation, strain distribution, bone stock

## Abstract

In total hip arthroplasty, excessive acetabular cup deformations and altered strain distribution in the adjacent bone are potential risk factors for implant loosening. Materials with reduced stiffness might alter the strain distribution less, whereas shell and liner deformations might increase. The purpose of our current computational study was to evaluate whether carbon fiber-reinforced poly-ether-ether-ketones with a Young´s modulus of 15 GPa (CFR-PEEK-15) and 23 GPa (CFR-PEEK-23) might be an alternative shell material compared to titanium in terms of shell and liner deformation, as well as strain distribution in the adjacent bone. Using a finite element analysis, the press-fit implantation of modular acetabular cups with shells made of titanium, CFR-PEEK-15 and CFR-PEEK-23 in a human hemi-pelvis model was simulated. Liners made of ceramic and polyethylene were simulated. Radial shell and liner deformations as well as strain distributions were analyzed. The shells made of CFR-PEEK-15 were deformed most (266.7 µm), followed by CFR-PEEK-23 (136.5 µm) and titanium (54.0 µm). Subsequently, the ceramic liners were radially deformed by up to 4.4 µm and the polyethylene liners up to 184.7 µm. The shell materials slightly influenced the strain distribution in the adjacent bone with CFR-PEEK, resulting in less strain in critical regions (<400 µm/m or >3000 µm/m) and more strain in bone building or sustaining regions (400 to 3000 µm/m), while the liner material only had a minor impact. The superior biomechanical properties of the acetabular shells made of CFR-PEEK could not be determined in our present study.

## 1. Introduction

The aseptic loosening of the acetabular cup due to stress shielding and altered strain distributions within the adjacent bone stock is a common cause for the failure of a total hip replacement [1]. If the modular cups are stiffer than the adjacent bone cavity, the strain distribution is altered, leading to high strains in some regions and low strains in others. It results in a potential risk of bone fracture on the one hand and bone atrophy on the other [2,3,4]. To avoid these effects, the stiffness of the acetabular cup should be similar to the adjacent bone [5]. Dickinson et al. investigated the influence of the material of monolithic acetabular cups on the pelvis cortex surface strains using a composite bone model. It was shown that monolithic cups made of ultra-high molecular weight polyethylene (UHMWPE) have less influence on the load transfer into the bone stock, when compared to metallic cups, as the reduced elastic modulus of UHMWPE bears more similarity to the bone stock [2]. In terms of the liner material, Kim et al. observed no significant differences in bone remodeling after five years postoperatively when ceramic and UHMWPE liners combined with structurally identical metallic shells were compared [6]. Therefore, the shell material of modular acetabular cups might have a stronger influence on the strain distribution than the liner material and shells made of polymers might have a lower influence on strain distribution.

Besides UHMWPE, poly-ether-ether-ketone (PEEK), often applied with carbon fibers for reinforcement (CFR-PEEK), is a biocompatible polymer that has been introduced as a material for monolithic cups and liners, but not as a material for acetabular shells [7,8,9,10,11].

However, PEEK and CFR-PEEK might be suitable alternatives to metal shells in modular acetabular cups as well, in order to avoid adverse effects on stress and strain distribution within the bone stock. However, it was shown that the reduced stiffness of shells made of PEEK and CFR-PEEK leads to increased shell deformations in the case of press-fit fixation in a finite element analysis (FEA) [12]. In particular, shells made of pure PEEK without reinforcement were deformed excessively, resulting in strong deformations of the liners, which might cause further problems in vivo. Excessive liner deformations can lead to a reduced clearance between the liner and femoral ball head and subsequently to increased frictional torques and increased wear rates [13,14,15,16]. Moreover, the seating of the liner might be jeopardized by strong shell deformations [17,18], which might lead to increased peak stresses within ceramic liners and, therefore, an increased risk of fracture [19,20,21]. Thus, shells made of PEEK without carbon fiber reinforcement do not seem to be a suitable alternative, whereas the suitability of shells made of CFR-PEEK needs to be further evaluated.

Hence, the aim of this computational study was to evaluate whether acetabular shells made of CFR-PEEK can serve as suitable alternatives to shells made of titanium in terms of strain distribution in the adjacent bone and whether the stiffness of such shells is sufficient to withstand adverse shell and liner deformations when they are pushed into an under-reamed bone cavity.

## 2. Materials and Methods

In the present numerical study, the insertion of a modular acetabular cup into a hemi-pelvis was simulated using ABAQUS/CAE (v 6.12-2, Dassault Systèmes Simulia Corp., Providence, RI, USA). The modular cup was designed based on a commercially available cup consisting of a titanium shell for press-fit fixation and a ceramic liner. The shell was flattened and spherical, with an outer diameter of 54 mm and a wall thickness of approximately 5.8 mm in the region of press-fit contact. The liner had an inner diameter of 36 mm and a maximum wall thickness of approximately 3.9 mm. In terms of simplification and comparability, the liner geometry was identical for both liner materials (ceramic and polyethylene), even so the real parts would have geometric differences in the dependency of the material. To apply realistic joint loads, a ceramic ball head was simulated in the form of an ideal sphere, with a diameter of 36 mm.

The hemi-pelvis included an acetabulum, which was equipped with a bone cavity, which was adapted to the shells outer diameter to simulate a diametric press-fit of 1 mm. The modular acetabular cup was oriented at 45° of inclination and 15° of anteversion, relative to the pelvic plane.

Due to the complex geometry, the hemi-pelvis was meshed using tetrahedral elements (141,877 elements), while the shell, liner, and ball head were meshed with 5987, 6162, and 14,400 hexahedral elements, respectively. To avoid excessive computational time, the components had to be meshed using linear elements. The element length at the contact area of the hemi-pelvis was set to 1.0 mm and the global element length of the acetabular shell, liner and head were set to 1.5 mm, 1.5 mm and 1.25 mm, respectively. A convergence analysis was carried out previously in order to determine the sensitivity of the results in relation to the mesh density, the element length of each component was varied between 1 mm and 4 mm. To exclude the influence of contact definitions on the results of the convergence analysis, each component was evaluated separately by means of deformation analyses. Thereby, the individual components were deformed by a defined amount and the deforming force was evaluated. The deforming forces determined with the chosen mesh densities changed less than 6% compared to the finest mesh.

The shell material was varied between a titanium alloy and two different kinds of CFR-PEEK (CFR-PEEK-15 and CFR-PEEK-23), while the liner material was varied between alumina toughened zirconia (ATZ) ceramic and UHMWPE. All the chosen materials were defined to be homogenous and isotropic (Table 1). As the yield strengths of the polymers were not exceeded, all the materials were simulated to be linear elastic.

The numerically simulated hemi-pelvis was reconstructed from computed tomography (CT) data of a human cadaveric hemi-pelvis (Ethics Committee of the University of Rostock; Reg. No. A 2009 38), using an algorithm introduced by Kluess et al. [23]. The local X-ray attenuation from the CT resulted in a distribution of Hounsfield units (HU) in the CT slices, which directly correlate with bone density and were therefore mapped onto the FE mesh using a previously described approach [24]. For this, the HU of the CT dataset were treated as temperatures of a temperature-dependent material model assigned to the FE nodes. This results in a realistic, heterogeneous distribution of material characteristics throughout the bone geometry. To assign a corresponding Young’s modulus to the HU, the HU from the CT were correlated with the local apparent densities, and the apparent densities were correlated with the Young’s modulus. Due to the fact that the existing CT data provided no scanned bone mineral density phantom, an apparent density of 1.8 g/cm^3^ was assigned to the maximal HU value, as per Taddei et al. [25]. The HU values of the cancellous bone were averaged, and the averaged value was assigned with an apparent density of 0.425 g/cm^3^, which represents a reasonable value for cancellous pelvic bone [26]. A linear correlation between the HU and apparent density was assumed using the following equation:

ρ_app_ (g/cm^3^) = 0.0007918*HU + 0.4718988 (1)

To calculate the Young’s modulus from the apparent density, the following equation based on the equation of Carter and Hayes was used [27]:

E (GPa) = 3.79e^0.06^ρ_app_^3^ (2)

Forty equally spaced reference points were selected between the maximum and minimum HU, at which the Young’s modulus was calculated and assigned. Between the reference points, a linear interpolation was used. Moreover, a lower limit of 500 MPa and an upper limit of 20,000 MPa were defined as thresholds to adequately represent the stiffness limits of the cancellous and cortical bones [28]. The mapping of the HU values and correlation to local stiffness was achieved with the self-developed software script AbaCTMat, based on Python 2.6.2 and implemented as plug-in in ABAQUS/CAE.

The model included three sliding contact formulations, which were described by normal and tangential contact. A penalty friction model with Coulomb friction formulation was chosen. The first contact was defined between the outer surface of the shell and the hemi-pelvis. The friction coefficient was set to 0.6, which represents a reasonable value for contacts between bones and porous coatings [29]. The second contact was defined between the shell and the liner, where a friction coefficient of 0.16 was applied [30]. The third contact was defined between the liner and the ball head, with a friction coefficient of 0.05 [13].

**Figure 1:**
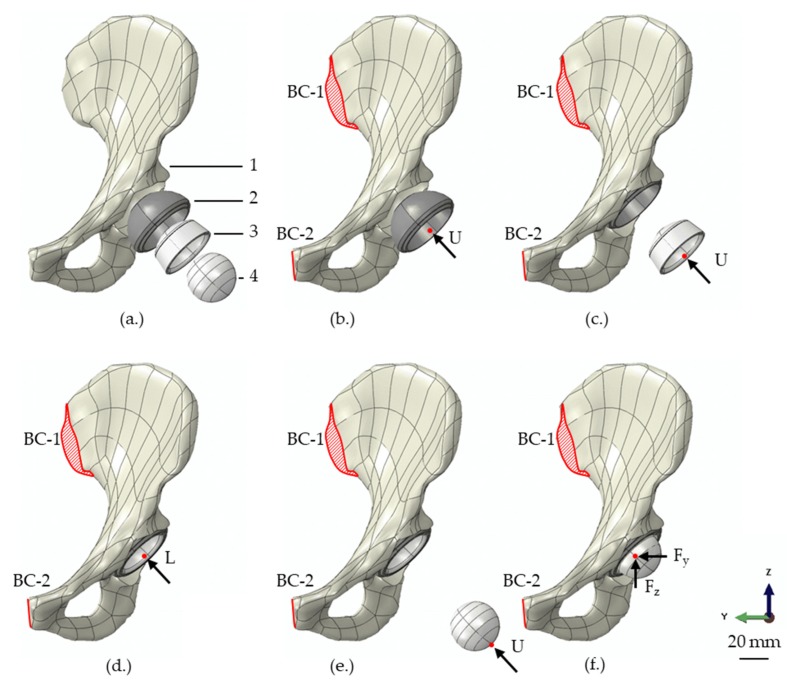
Depiction of the assembly (**a**) of the 3D finite element model consisting of a hemi-pelvis with a prepared bone cavity (1), acetabular shell (2), liner (3) and femoral ball head (4). The simulation was executed in several steps, consisting of the displacement-controlled insertion of the shell (**b**), displacement-controlled motion of the liner until the first contact to the shell (**c**), force-controlled insertion of the liner (**d**), displacement-controlled insertion of the femoral ball head (**e**) and the final loading, by applying a realistic hip joint load in x-, y- and z-direction (F_x_, F_y_ and F_z_) via the femoral ball head (**f**). During all simulation steps, the translational degrees of freedom at the sacroiliac joint were completely fixed (BC-1) and the pubic symphysis was fixed in the y-direction (BC-2).

The simulation was executed in subsequent steps. Initially, the constraints for the hemi-pelvis were applied at the sacroiliac joint and the pubic symphysis, whereby the translational degrees of freedom at the sacroiliac joint were completely fixed (BC-1), while the motion in the sagittal plane was enabled at the pubic symphysis (BC-2) [13] (Figure 1). In the first step, the acetabular shell was pushed under displacement-control into the cavity, until a predefined shell overhang was reached using a kinematic coupling definition (Figure 1b). In the following step no forces or displacements were applied, to enable an elastic relaxation of the shell and cavity. Thereafter, the liner was also moved in a displacement-controlled manner, until a first contact between the shell rim and the liner was achieved (Figure 1c), followed by a force-controlled insertion with 500 N using a kinematic coupling definition again (Figure 1d). The coupling was applied to the distal inner surface of the liner, to not bias the radial deformation. Subsequently, the femoral ball head was inserted in a displacement-controlled manner into the liner to initiate contact (Figure 1e), followed by the application of a realistic hip joint load (Figure 1f). A load during normal walking (resulting load: 784.8 N) was chosen in accordance with Bergmann et al. [31] and applied via a reference point to the ball head using a kinematic coupling, again. The load was divided into the different load compounds in the x-, y-, and z-directions (F_x_ = 502.3 N, F_y_ = −78.5 N and F_z_ = 2048.3 N).

The numerical simulations were performed as non-linear static calculations with incremental loading conditions. The FEA was evaluated in terms of the maximum radial shell and liner deformation after insertion and subsequent relaxation. Therefore, polar coordinate systems were created along the rotational axis of the shell and liner, respectively, and circumferential node paths along the inner rim of the acetabular shell (62 nodes) and liner (47 nodes) were defined and evaluated.

**Figure 2:**
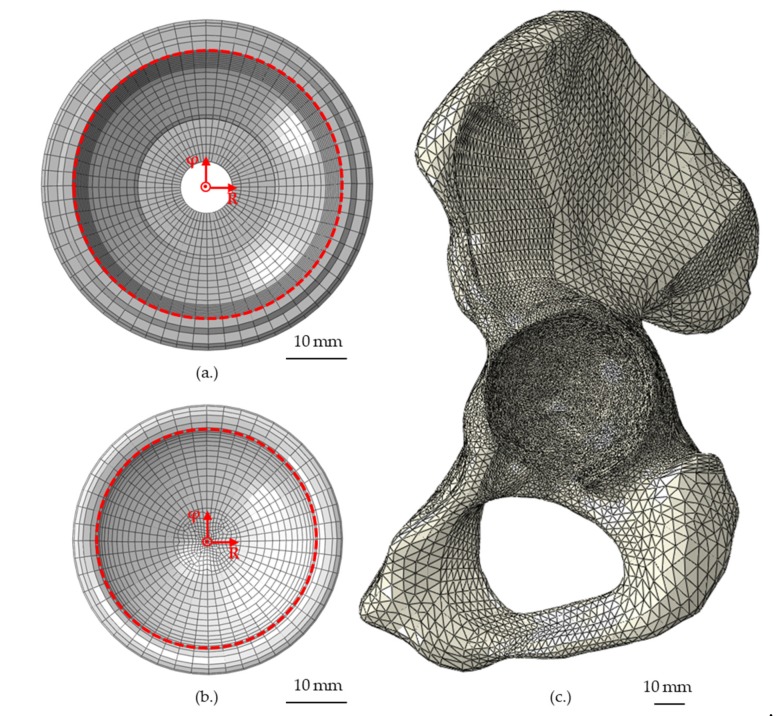
Depiction of the node paths at the inner rim of the shell (**a**) and liner (**b**), consisting of 62 and 47 nodes, to analyze the radial deformation in polar coordinate systems. Moreover, the analyzed node region in terms of strain distribution at the surface of the hemi-pelvis is depicted (**c**).

Moreover, the strain distribution over the full surface of the hemi-pelvis was investigated at a total of 29,923 node points (Figure 2c). It should be noted that only the magnitudes of the strains were considered and therefore no distinction was made between the compression and tensile strains.

The determined logarithmic strains were divided into different strain regions, dependent on the bone response, based on studies by Biewener [32] and Frost [33], as summarized in Table 2.

## 3. Results

### 3.1. Shell and Liner Deformation

**Figure 3:**
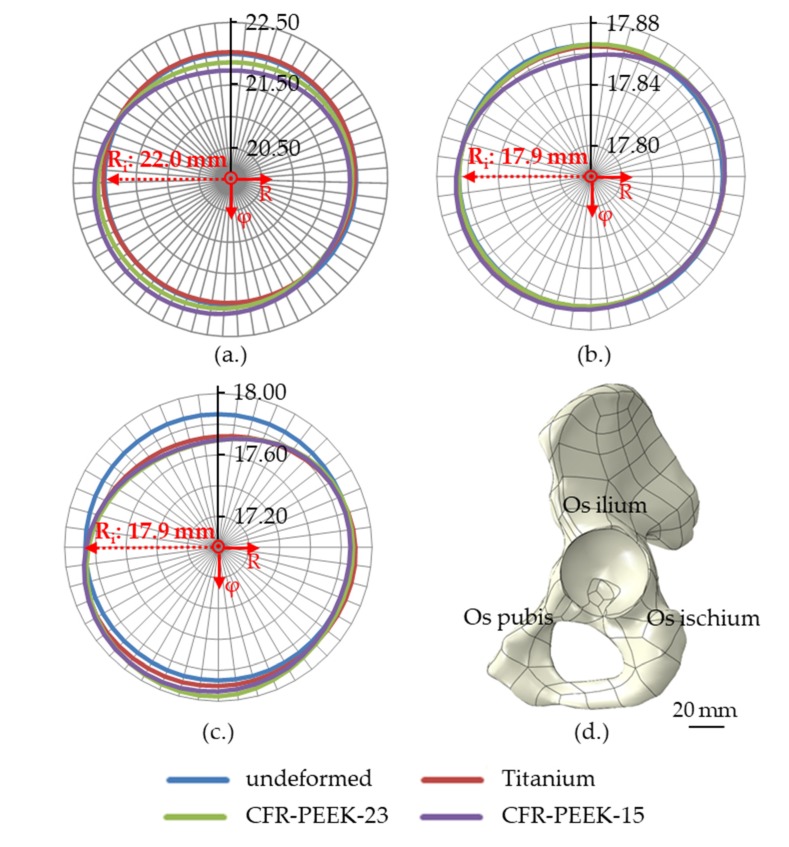
Exaggerated radial deformation (mm) at the inner rim of the shell (**a**) and liners made of ATZ ceramic (**b**) and UHMWPE (**c**) dependent on the chosen shell material and the orientation of the hemi-pelvis (**d**). The deformations are depicted in a polar coordinate system and in relation to the initial radii (R_i_).

After the insertion into the hemi-pelvis and subsequent relaxation of the shells, the shell made of CFR-PEEK-15 showed the highest deformation (266.7 µm), followed by the shell made of CFR-PEEK-23 (136.5 µm) and the shell made of titanium, which exhibited the smallest maximum deformation (54.0 µm). Therefore, the deformation of shells made of CFR-PEEK-15 was approx. two and five times the deformation of shells made of CFR-PEEK-23 and titanium, respectively, while the deformation of a shell made of CFR-PEEK-23 was approx. 2.5 times higher than the deformation of the titanium shell. The shells made of CFR-PEEK were compressed in the region of the Os ilium and Os ischium and expanded in the direction of the acetabular notch (Figure 3a).

The deformation of the shells influenced the radial deformation of the liners, with the largest liner deformation resulting in combination with the shell made of CFR-PEEK-15, followed by CFR-PEEK-23. The UHMWPE liners displayed greater deformation compared to ceramic liners. The maximum radial liner deformations have been summarized in Table 3.

Like the shells, the liners were compressed in the region of the Os ilium and Os ischium and expanded in the direction of the acetabular notch (Figure 3b,c).

### 3.2. Strain Distribution

The resulting strains in the hemi-pelvis were influenced by the shell material (Table 4). The strongest influence could be seen for strains beneath 3000 µm/m. The percentage of nodes in the region of expected bone loss (<400 µm/m) was higher in shells made of CFR-PEEK, while the percentage of nodes in a region of bone preservation and building (400–3000 µm/m) was lower. On the other hand, the percentage of nodes with strains above 20,000 µm/m was higher in shells made of titanium.

**Figure 4:**
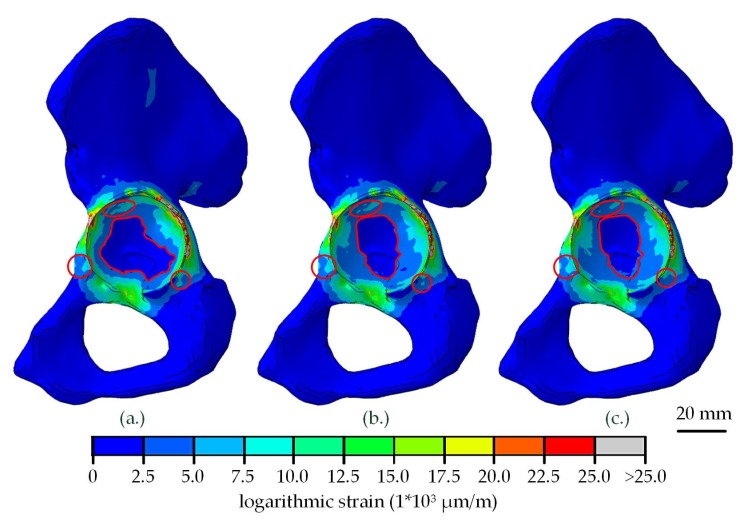
Strain distribution in the adjacent bone of the hemi-pelvis after relaxation of the acetabular shell, dependent on the chosen shell material (**a**) titanium, (**b**) CFR-PEEK-23 and (**c**) CFR-PEEK-15. Differences visible on the macroscopic scale are highlighted in red.

On the macroscopic scale, there were almost no differences of the visible strain distribution (Figure 4). The strongest alteration of the strain was seen at the acetabular fossa, where the strain increased when CFR-PEEK was used instead of titanium. Minor deviations were seen at the Os ischium and Os pubis.

**Figure 5:**
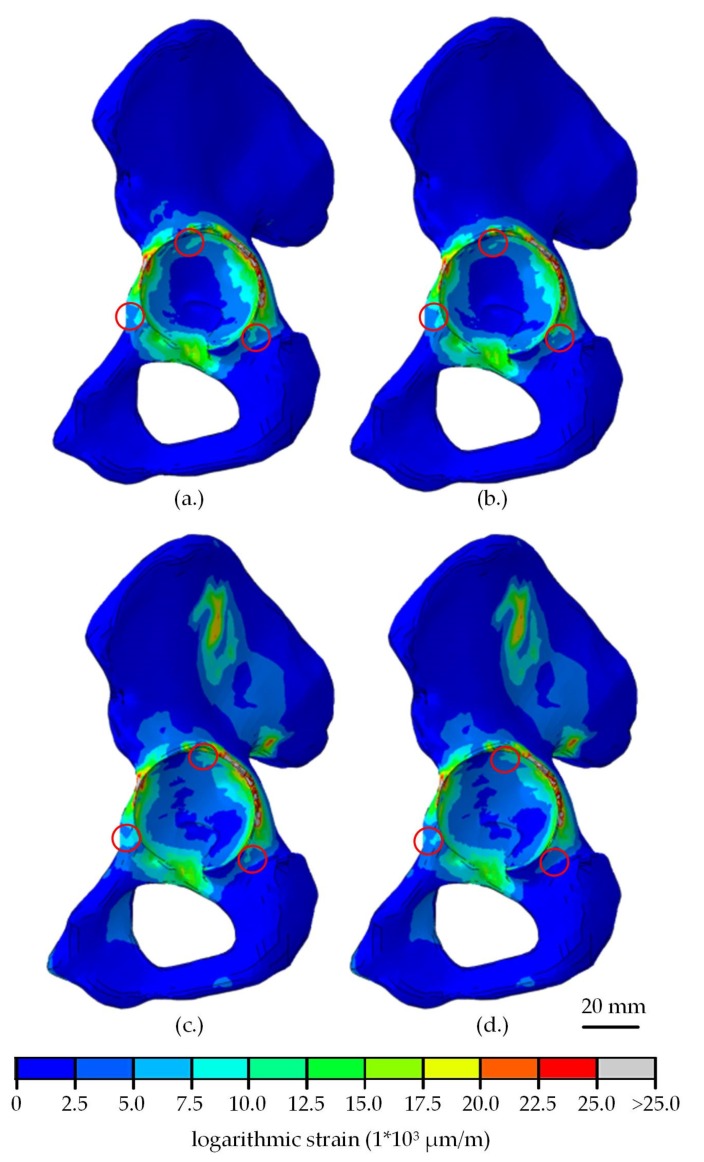
Comparison of strain distribution in the adjacent bone of the hemi-pelvis after the UHMWPE liner insertion into the titanium (**a**) and CFR-PEEK-15 (**b**) shell, followed by a relaxation. Additional the alteration of strain distribution after the application of a hip joint load in dependency of the shell material (**c**) titanium, (**d**) CFR-PEEK-15.

The liner material had a small influence on the strains in the hemi-pelvis after insertion and subsequent relaxation, with more nodes with a strain beneath 400 µm/m and less nodes between 400 µm/m and 20,000 µm/m, when UHMWPE was used (Table 5). Due to the joint load, the overall strains increased and the influence of the liner material was compensated (Table 5 and Figure 5). The percentage of nodes with strains beneath 400 µm/m was reduced up to 73.0%, due to the hip load, while the percentage of nodes with strains in the range from 400 µm/m to 3000 µm/m and from 3000 µm to 20,000 µm/m increased up to 21.0% and 19.8%, respectively.

After the insertion of the liner, no relevant differences in strain distribution in the dependency of the shell or liner material were visible on the macroscopic scale. Only small areas near the acetabular rim showed altered strain distribution due to the shell material (Figure 5a,b). After loading with a hip joint load, the strains in the acetabulum and the wing of ilium increased strongly (Figure 5c,d). A small effect of the shell material was seen in the same regions as in the unloaded condition. In Figure 5, only the shells made of titanium and CFR-PEEK-15 with a liner made of UHMWPE were compared as an example. The results of the other simulations showed similar strain patterns. 

## 4. Discussion

In total hip arthroplasty, the stiffness of modular acetabular cups should be similiar to the adjacent bone, to avoid stress and strain shielding, that could otherwise lead to subsequent atrophy in the adjacent bone [5,34]. Compared to titanium, polymer materials are characterized by Young’s modulus closer to bone and, therefore, might alter the stress and strain distribution to a lesser degree. Particularly, reinforced polymers, such as CFR-PEEK, might be a suitable alternative for acetabular cups, as their material properties can be altered to fit the bones’ properties by varying the proportion of carbon fibers. However, the reduced stiffness compared to titanium shells might lead to increased acetabular shell and liner deformation, that can subsequently lead to a reduced clearance, possibly resulting in higher frictional torques and wear rates [13,14,15,16,19,20].

CFR-PEEK has previously been tested in numerical, experimental and in vivo studies as a material for monolithic cups and liners, but barely as a shell material [7,8,9,10,11,35,36]. Therefore, the purpose of our current computational study was to evaluate whether the stiffness of two different CFR-PEEK materials is sufficient to avoid excessive shell and liner deformation when they are used as shell materials for modular acetabular cups and whether these shells may reduce the strains in the adjacent bone.

Our study is restricted by certain limitations. First of all, only one hemi-pelvis was used in the current study and, thus, anatomical differences and differences in terms of age-related bone properties could not be taken into account. In particular, the bone density might be crucial in terms of shell deformation, even though previous studies concluded that altered bone properties do not influence the stress distribution greatly [36]. Moreover, the hemi-pelvis was CT-scanned without an additional bone mineral density phantom. Therefore, the equation to correlate the HU and apparent density had to be set manually, using additional data from the literature [25,26]. To calculate the Young’s modulus from the apparent density, an equation based on an equation by Carter and Hayes [27], which was originally set up to calculate the compressive modulus depending on the apparent density and strain rate, was used as this equation can be used for cancellous as well as cortical bones. The numerically simulated hemi-pelvis showed unreasonably small HU in a region in the Os ilium; therefore, a minimum Young’s modulus of 500 MPa was established, to avoid areas with excessively low modulus. The maximum Young’s modulus of the cortical bone was set to 20,000 MPa, which is high compared to other studies, in which the maximal Young’s modulus for cortical bones was set to between 8500 MPa and 18,000 MPa [20,23,36,37]. This is applicable, because the highest HU was defined as the point of maximum stiffness, but this point still includes naturally occurring deviations in x-ray attenuation which, however, are compensated over several HU. This still may lead to a slight overestimation of the bone stiffness, but is favored in order to consider a disadvantageous scenario in terms of acetabular shell and liner deformations. Moreover, these deformations were not experimentally validated in the current study, as the simulated hemi-pelvis was not accessible for experimental analysis. It is assumed that a qualitative comparison between the shells is legitimate, as the evaluated feature for shell and liner deformation, as well as strain distribution, was the material definition of the shell. Additionally, the material behavior of the CFR-PEEK was simplified. The CFR-PEEK was simulated as elastic with isotropic behavior, despite CFR-PEEK being an anisotropic material with its mechanical properties depending on the carbon fiber orientation. By adjusting the fiber orientation in the direction of strongly loaded areas, the shell could be strengthened, possibly leading to reduced shell deformations in these areas. Hence, an advanced material definition should be considered for future investigations. Moreover, it is known that implants made of CFR-PEEK have to be coated to enable osseointegration in vivo [38,39,40]. Even so, it was previously shown that the coating procedure can influence the mechanical properties (e.g., yield strength) of PEEK materials [41], the material properties of uncoated CFR-PEEK were used in the current study in terms of simplification.

Despite these limitations, the current study was suitable for providing an insight into whether CFR-PEEK can be used as an alternative shell material in the context of acetabular shell and liner deformation, as well as strain distribution in the adjacent bone. In terms of shell deformation, a correlation between the shell material stiffness and deformation value was determined, as shown previously [12]. The determined maximum radial deformation of the titanium shell after insertion into the bone cavity was 54.0 µm. When the shells made of CFR-PEEK-23 or CFR-PEEK-15 with a reduced stiffness were simulated, the radial shell deformation increased by about 2.5 times and 5 times, respectively. In a previous study, the same shell was numerically inserted into bone substitute cavities, with a diametric press-fit of 2 mm. The deformation of the titanium shell in the present study is about three times, and the deformation of the shell made of CFR-PEEK-23 is still about 1.5 times higher compared to the data of the previous study, even so the diametric press-fit was only 1 mm, indicating that the simulated hemi-pelvis is stiffer compared to the bone substitute material used before [12]. In another study, Everitt et al. inserted monolithic cups made of CFR-PEEK with a Young’s modulus of 15 GPa into cavities made of polyurethane foams (20 pcf or 30 pcf), with a diametric press-fit of 1 mm or 2 mm [42]. The monolithic cup was deformed by about 250 µm when inserted into a 1 mm under-reamed cavity made of 20 pcf polyurethane foams, which is in good agreement with the present deformation in the case of the shell made of CFR-PEEK-15. When the monolithic cups were inserted in 2 mm under-reamed cavities or cavities made of 30 pcf polyurethane foams, the deformation doubled [42]. In case of modular cups, an increased shell deformation results in greater liner deformations and stresses, harboring the risk of reduced clearance, increased frictional torques, incomplete seating and misalignment, as well as higher wear rates at the articulating surfaces [13,14,15,16]. Therefore, a diametric press-fit above 1 mm or the insertion of acetabular shells made of CFR-PEEK in young patients with high bone mineral density could be critical in terms of deformation, and the applicability of CFR-PEEK shells has to be cautiously questioned in these cases. In further investigations, the effect of an increased press-fit and increased bone mineral density on the implant deformation should be considered. On the other hand, one advantage of CFR-PEEK is the ability to adjust the material properties for a specific application. For example, the orientation of the carbon fibers could be altered to create an anisotropic material that can be used for acetabular shells. It would be conceivable to radially reinforce the shell in order to avoid strong deformations, while remaining flexible in the direction of the load application. Another opportunity to alter the mechanical properties of PEEK is by adding various additives (like TiO2) to the PEEK powder before molding, which can increase the bioactivity and therefore osseointegration at the same time [43].

The shells were mainly compressed in the contact area at the Os ilium and Os ischium, and expanded in the direction of the Os pubis and acetabular notch. Therefore, the shell deformation was consistent with the distribution of the bone density, as the deformation was initiated from the stiffest region. This is also in line with previous studies, in which similar deformation patterns were found [13,20,44]. In the present study, only the initial deformation of the shells was determined, even though creep deformation over time is a well-known problem of polymers. The creep deformation of shells made of polymers might lead to liner migration and, subsequently, to altered liner deformations and stresses. In worst-case scenarios, this could lead to increased rates of ceramic liner fractures, as reported for sandwich liners [45,46,47,48,49,50,51]. Therefore, the creep deformation of acetabular shells made of CFR-PEEK should be considered in future long-term experimental investigations.

The radial liner deformation in the present study was in a range between 149.0 µm (titanium) and 184.7 µm (CFR-PEEK-23) in case of the UHMWPE liner, and 1.8 µm (CFR-PEEK-23) and 4.4 mm (CFR-PEEK-15) in case of the ceramic liner after the load-controlled insertion and subsequent relaxation. Therefore, a linear correlation between shell and liner deformation was not shown, although previous studies have shown that increased shell deformations lead to the increased deformation of the inserted liners [12,13,46]. The non-uniform liner deformations might occur due to other factors, like the liner’s initial seating depth after load-controlled insertion, and a varying bounce-back after the liner relief.

Compared to the above mentioned study [12] in which the same acetabular cups were numerically inserted into bone substitute cavities, the deformation of the UHMWPE liner was deformed six times more in case of the titanium shell and 1.5 times in case of the CFR-PEEK-23 shell during the load-controlled liner insertion. After subsequent relaxation, the liner deformation decreased, but was still three times and 1.5 times higher, respectively. However, when the determined liner deformations are compared to typical radial clearance of 300 µm for hard-on-soft bearings [52], the articulation between a femoral ball head and an UHMWPE liner would not be hampered in combination with the simulated shell materials. The deformations of the ceramic liner were also influenced by the chosen shell material, but were less critical regarding the clearance. The maximum radial deformation of the ceramic liner ranged from 1.8–4.4 µm and, thus, was approximately 20% of a typical radial clearance for ceramic-on-ceramic bearings of 20 µm [53]. The maximum determined peak tensile stress of the ceramic liner (129.9 MPa), arising during load-controlled liner insertion, was nowhere near a critical value in terms of ceramic fracture as well [54]. However, under unfavorable conditions, such as an increased press-fit, a decreased wall thickness of the shell, a high bone mineral density, or increased loads, the radial deformation of the shell would increase, leading to increased liner deformation, increased stresses and a further decrease of the clearance, especially in the case of the UHMWPE liners [12,13,14,17,18,42,44,55,56,57]. Decreased clearances are known to increase the wear rates [15] and frictional torques at the articulating surfaces and therefore, might hamper the initial fixation of acetabular cups [16].

The magnitudes of the strains in the hemi-pelvis were slightly influenced by the stiffness of the shell material. With the decreasing stiffness of the shells, the proportion of nodes with a strain of less than 400 µm/m increased, indicating that more nodes are located in a bone resorbing range in the case of shells made of CFR-PEEK as compared to titanium, with the most affected region detected at the acetabular fossa. At the regions where the shell is in direct contact with the bone, the highest strains occurred (overloading), indicating a possible risk of bone fracture during shell insertion. In these regions, the strains were the lowest when the acetabular shell made of CFR-PEEK-15 was simulated. However, it is assumed that the determined strains would be reduced in vivo over time, due to the viscoelastic response of the adjacent bone stock, allowing the re-expansion of the shells [14,56].

Overall, only small effects of the shell material on the strains were determined, supporting the conclusions of other studies that a simple change in material stiffness is not sufficient to avoid the loss of acetabular bone [36,58]. The chosen liner material had nearly no impact on the strain distribution under loading, which is also in line with the findings of other studies [6,59,60].

The strain distribution pattern at the surface of the bone cavity was similar to previous studies [36,59]. The peak strains occurred at the rim of the reamed bone cavity, and there was nearly no strain at the acetabular fossa, as shown previously [36,59,61]. In our present FE study, only the magnitudes were evaluated. We found that the strains in the acetabulum were mainly tensile strains, except for the surfaces in direct contact to the shell, where compressive strains arose.

In the present study, only three loading states were analyzed: either directly after the shell and liner insertion with subsequent relaxation (no external loading), or at the peak load of a normal gait cycle [31,62]. We showed that nodes in the bone sustaining or building region of 400–3000 µm/m increased up to 21.0% during the loading, compared to the unloaded cup. Therefore, the loading condition was crucial in terms of strain distribution, as expected, and more loading conditions, such as stair-climbing, sitting down and standing up, or stumbling, with higher loads and altered loading directions should be considered in further investigations [63].

## 5. Conclusions

In conclusion, the deformation of acetabular shells made of CFR-PEEK is more pronounced as compared to titanium shells during intraoperative insertion, resulting in the increased radial deformation of liners. However, liner deformations were not critical in terms of the radial clearance in our present computational study. Particularly, the combination of shells made of CFR-PEEK and liners made of ATZ ceramic showed only minor differences in implant deformations, as compared to titanium shells. In terms of strain distribution, only small effects were observed to be dependent on the shell and liner material, with a greater area of bone being in a bone-sustaining or building region when CFR-PEEK was used. Therefore, clear advantages in terms of strain distribution were not seen in our computational study. Acetabular shells made of CFR-PEEK are not likely to replace titanium shells in case of standard THA. However, as the strain distribution was not impaired and the shell and liner deformations were not critical, shells made of CFR-PEEK might be a possible alternative in special cases. However, in our present study only shell and liner deformations as well as strain distribution in the adjacent bone were considered, but even so, more parameters have to be taken into account before a final statement can be made about the suitability of acetabular shells made of CFR-PEEK. Therefore, further experimental investigations are needed, e.g., to determine the creep and wear properties of acetabular shells made of CFR-PEEK.

## Figures and Tables

**Table 1:** Overview of the Young’s modulus and Poisson’s ratio defined for the various simulated materials.

Part	Material	Young’s Modulus (MPa)	Poisson’s Ratio
Acetabular shell	Titanium	110,000	0.40
	Carbon fiber-reinforced poly-ether-ether-ketone (CFR-PEEK-15)	15,000	0.40
	Carbon fiber-reinforced poly-ether-ether-ketone (CFR-PEEK-23)	23,000	0.40
Liner	Alumina toughened zirconia (ATZ) ceramic	261,000	0.27
	Ultra-high-molecular-weight polyethylene (UHMW-PE) [22]	945	0.45
Ball head	Alumina toughened zirconia (ATZ) ceramic	261,000	0.27

**Table 2:** Values set for bone thresholds and response to particular strain rates [32,33].

Strain (µm/m)	Bone Response
<400	Atrophy
400–3000	Bone preserving and building
3000–20,000	Yielding
>20,000	Fracture

**Table 3:** Maximum radial liner deformations dependent on the acetabular shell and liner material.

Shell Material	Titanium		CFR-PEEK-23		CFR-PEEK-15	
Liner material	Ceramic	UHMWPE	Ceramic	UHMWPE	Ceramic	UHMWPE
Maximum deformation (µm)	2.0	149.0	1.8	184.7	4.4	180.6

**Table 4:** Comparison of the percentage of analyzed nodes, categorized into the different strain regions after shell relaxation and dependent on the shell material (Titanium, CFR-PEEK-23 or CFR-PEEK-15).

Strain Region (µm/m)	Titanium	CFR-PEEK-23	CFR-PEEK-15
<400 (Atrophy)	17.2%	18.2%	18.3%
400 < x < 3000 (Bone preserving and building)	40.6%	39.7%	39.5%
3000 < x < 20,000 (Yielding)	39.8%	40.2%	40.5%
>20,000 (Fracture)	2.4%	1.8%	1.7%

**Table 5:** Comparison of the percentage of analyzed nodes, categorized into the different strain regions after liner insertion and after loading in dependency of the acetabular shell and liner material.

Strain Regions (µm/m)	Load Scenario	Titanium		CFR-PEEK-23		CFR-PEEK-15	
		Ceramic	UHMWPE	Ceramic	UHMWPE	Ceramic	UHMWPE
<400 (Atrophy)	Liner Insertion	17.3%	19.3%	18.9%	20.2%	19.3%	20.6%
	Loading	5.3%	5.3%	5.6%	5.5%	5.4%	5.6%
400 < x < 3000 (Bone preserving and building)	Liner Insertion	41.1%	39.1%	41.2%	39.9%	41.3%	40.8%
	Loading	47.2%	47.3%	48.5%	48.1%	48.0%	48.5%
3000 < x < 20,000 (Yielding)	Liner Insertion	39.3%	39.3%	38.1%	38.0%	37.7%	36.9%
	Loading	45.2%	45.1%	44.1%	44.5%	44.7%	44.2%
>20,000 (Fractures)	Liner Insertion	2.3%	2.3%	1.8%	1.9%	1.7%	1.7%
	Loading	2.3%	2.3%	1.8%	1.9%	1.9%	1.8%
